# Heterologous booster vaccination with CoronaVac following prime vaccination with mRNA vaccine

**DOI:** 10.1002/cti2.1449

**Published:** 2023-04-18

Yun Shan Goh, Siew‐Wai Fong, Angeline Rouers, Zi Wei Chang, Matthew Zirui Tay, Jean‐Marc Chavatte, Nicole Ziyi Zhuo, Pei Xiang Hor, Chiew Yee Loh, Yuling Huang, Joel Xu En Wong, Yong Jie Tan, Daniel Rui Xiang Lim, Bei Wang, Eve Zi Xian Ngoh, Siti Nazihah Mohd Salleh, Raphael Tze Chuen Lee, Surinder Pada, Louisa Jin Sun, Desmond Luan Seng Ong, Jyoti Somani, Eng Sing Lee, NCID Study Group, COVID‐19 Study Group, Sebastian Maurer‐Stroh, Cheng‐I Wang, Yee‐Sin Leo, Raymond TP Lin, Ee Chee Ren, David C Lye, Barnaby Edward Young, Poh Lian Lim, Lisa FP Ng & Laurent Renia

*Clinical & Translational Immunology* 2023; **12:** e1449.

**Correction to:** Clin Trans Immunol 2022; 11: e1043. https://doi.org/10.1002/cti2.1403. Published online 23 August 2022

The authors inadvertently excluded a funding body in the Acknowledgments section. The corrected version appears below:

